# Supplementary material for: Evolution of connectivity architecture in the Drosophila mushroom body
Source: Nat Commun. 2024 Jun 7;15:4872. doi: 10.1038/s41467-024-48839-4 (PMC11161526; doi:10.1038/s41467-024-48839-4)
Supplement: Supplementary file 1 — Supplementary Information [file 41467_2024_48839_MOESM1_ESM.pdf]

Supplementary Information

Evolution of connectivity architecture in the Drosophila mushroom body

by

Kaitlyn Elizabeth Ellis[1], Sven Bervoets[1], Hayley Smihula[1], Ishani Ganguly[2], Eva Vigato[1], Thomas O. Auer[3, 4], Richard Benton[3], Ashok Litwin-Kumar[2] and Sophie Jeanne Cécile Caron[1,*]

[1]School of Biological Sciences, University of Utah, Salt Lake City, United States.
[2]Center for Theoretical Neuroscience, Columbia University, New York, United States.
[3]Center for Integrative Genomics, Faculty of Biology and Medicine, University of Lausanne, Lausanne, Switzerland
[4]Present address: Department of Biology, University of Fribourg, Fribourg, Switzerland
[*]Correspondence: sophie.caron@utah.edu

Supplementary Table 1 | Morphological features of dye-labeled projection neurons across species.

Morphological features	DL2d projection neurons			DC3 projection neurons			VM5v projection neurons		
	D. melanogaster	*D. simulans*	*D. sechellia*	*D. melanogaster*	*D. simulans*	*D. sechellia*	*D. melanogaster*	*D. simulans*	*D. sechellia*
Bouton cluster volume (μm³)	89.65 ± 51.87	77.09 ± 33.41	141.54 ± 58.24	184.15 ± 34.60	169.01 ± 43.23	165.36 ± 25.56	116.45 ± 18.68	85.38 ± 17.48	169.73 ± 39.67
Primary axonal branch number	1 ± 0.70	2 ± 0.70	3 ± 1.27	2 ± 0.40	2 ± 0.49	2 ± 0.75	3 ± 0.70	4 ± 0.50	4 ± 1.20
Primary axonal branch length (μm)	29.84 ± 15.14	21 ± 9.18	35.08 ± 19.31	28.76 ± 15.10	35.96 ± 18.17	33.37 ± 15.99	26.46 ± 7.17	33.92 ± 13.68	20.84 ± 12.13
Axonal fork points	2 ± 0.80	2 ± 0.98	3 ± 1.02	3 ± 0.75	3 ± 0.75	2 ± 0.40	3 ± 0.75	4 ± 0.80	4 ± 1.02

Supplementary Table 1. Morphological features of dye-labeled projection neurons across species. Morphological features of individual DC3, DL2d and VM5v projection neurons — namely the presynaptic bouton volume, the number of axonal primary branches and their length, and the number of forks they form in the mushroom body — were measured and compared across species (*n* = 5 for DC3 neurons, *n* = 10 for DL2d neurons and *n* = 5 for VM5d neurons, standard deviation from mean is shown). See Figure 4b for representative images of each projection neuron type. All source data used in this table are provided in the Source Data file.

Supplementary Table 2

Odors used	Glomeruli activated	Cumulative connectivity frequencies (%)		
		D. melanogaster	***D. simulans***	***D. sechellia***
2,3 butanedione	DA4l, DC4, DM1, DM4, DP1l, DP1m, VA2, VM3, VM5d, VM7d	17.7	16.4	22.5
3-octanol	D, DC1, DC2, DM2, DM3, DM6, VA4, VC3, VM5d, VM5v	24.5	28.5	19.1
4-methylcyclohexanol	D, DA2, DM2, VA3	7.7	8.9	7.8
Farnesol	DC3	4.5	5	1.4
Hexanoic acid	DL2d (*D.sec*), DM2 (*D.mel, D.sim, D.sec*), VL2a (*D.mel*)	6.7	2.7	5.9
Isopentyl acetate	DA4l, DL1, DM2, DM3, DM6, VA4, VC1, VM3, VM5d, VM5v, VM7d	24.5	25.7	22.2

Supplementary Table 2. Connectivity frequencies and performance indices. Based on previous studies[21,33], glomeruli were determined to be activated by a particular odor if that odor can elicit at least 0.25 of the maximal possible response in the olfactory sensory neurons associated with that glomerulus. Cumulative frequencies were calculated by adding the connectivity frequencies measured for each of the glomeruli activated by a given odor in each species.

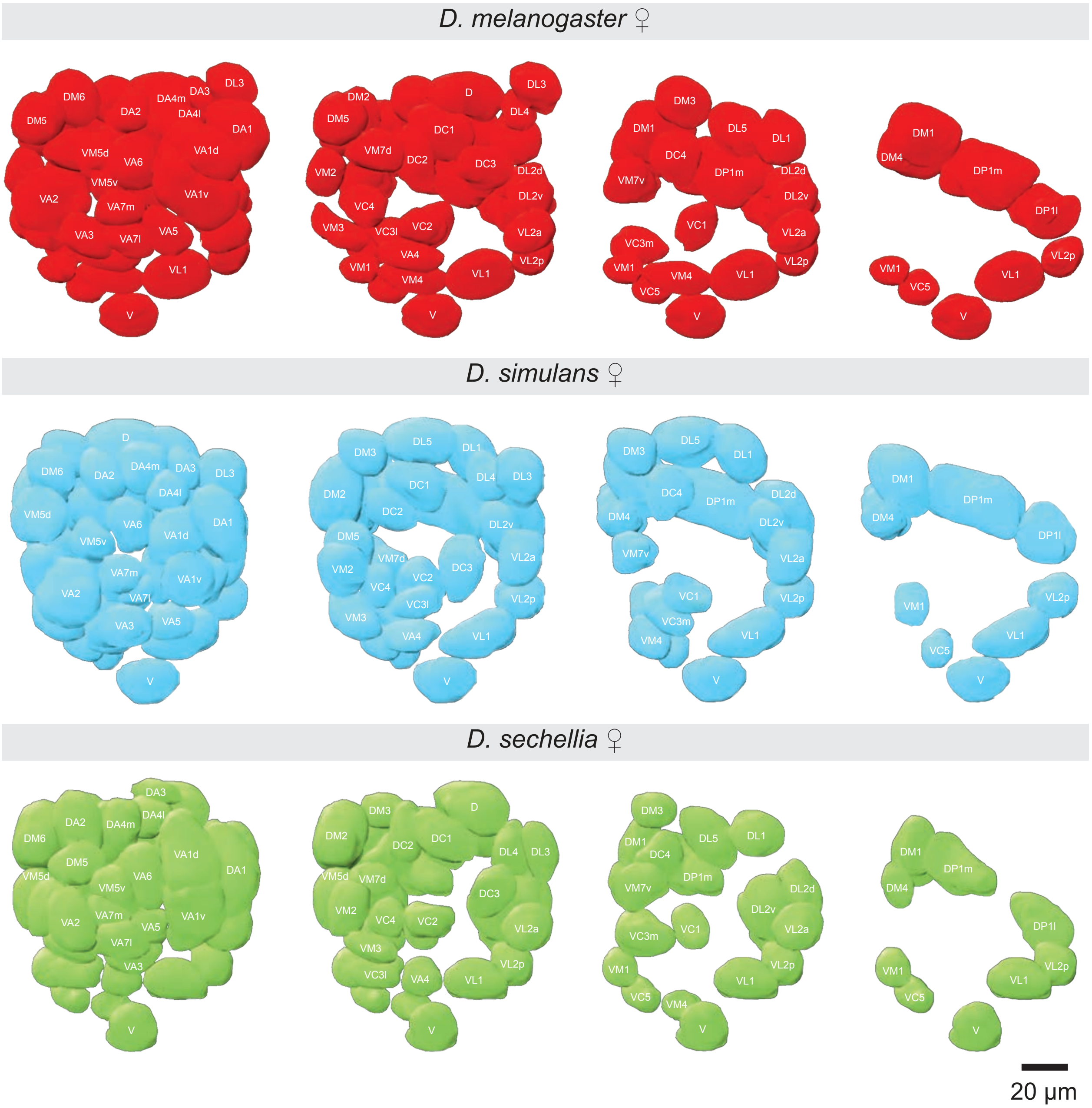

Supplementary Figure 1

Supplementary Figure 1. Antennal lobe reconstructions in different species. The brains of two- or three-day old *D. melanogaster* (red), *D. simulans* (blue), and *D. sechellia* (green) female flies were fixed, immuno-stained (using the nc82 monoclonal antibody against Bruchpilot) and imaged. The glomeruli forming the antennal lobe were individually reconstructed and identified based on shape and location. Three different planes are shown for each reconstruction. Scale bar is 20 μm. See Supplementary Table 1 for quantifications. All source data used in this figure are provided in the Source Data file.

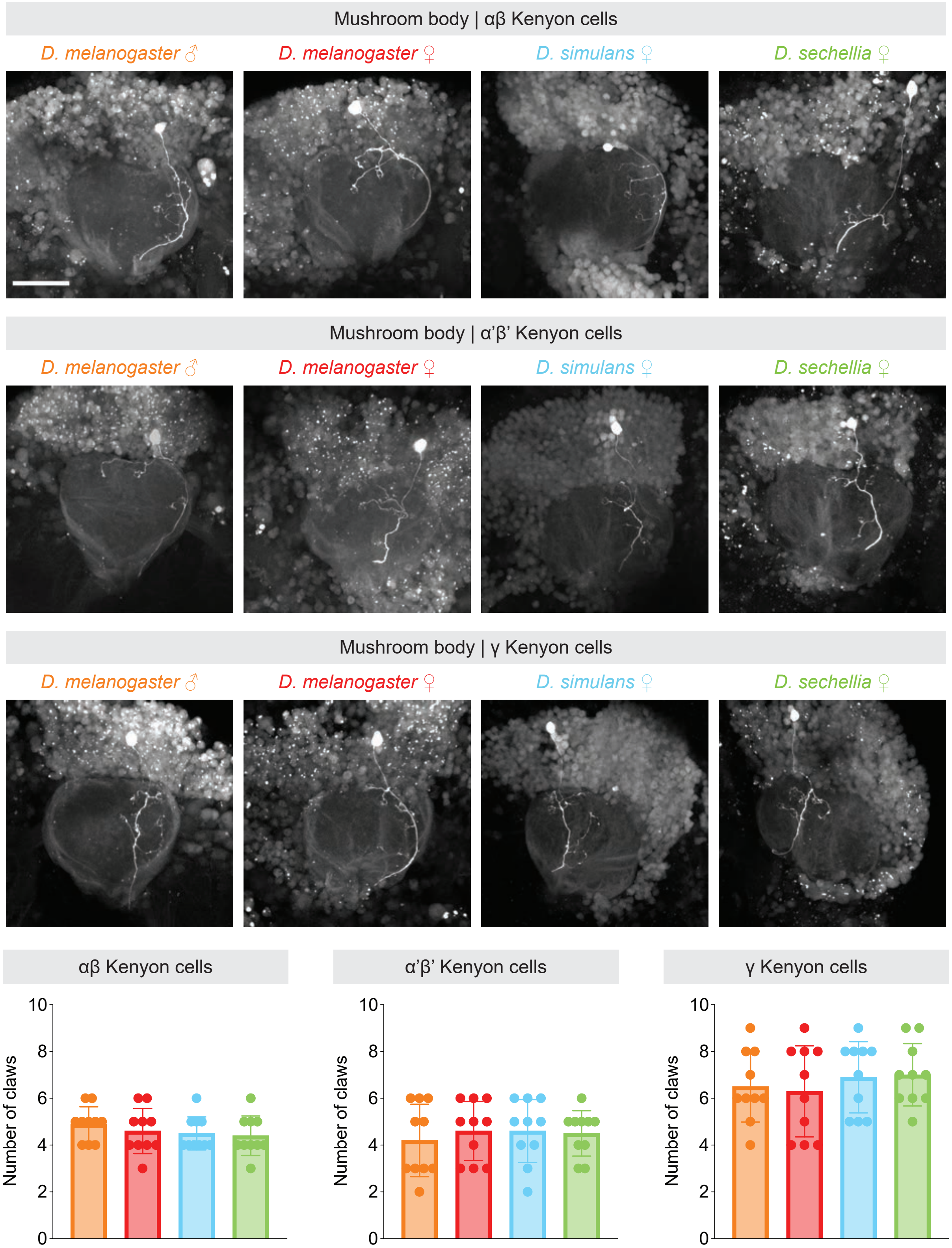

Supplementary Figure 2

Supplementary Figure 2. Morphological features of Kenyon cells across species. Individual α/β (top panels), α'/β' (middle panels), and γ Kenyon cells (bottom panels) were photo-labeled in *D. melanogaster* males (first column), *D. melanogaster* females (second column) *D. simulans* (third column), and *D. sechellia* flies (fourth column), and the postsynaptic terminals formed by these neurons in the mushroom body calyx — called claws — were imaged. Scale bar is 50 μm. The total number of claws per Kenyon cell were counted for different types of Kenyon cell (orange: *D. melanogaster* males; red: *D. melanogaster* females; blue: *D. simulans*; green: *D. sechellia*; n = 10, standard deviation from mean is shown). The statistical significance, or p-value, was measured to compare the number of claws measured for a given type of Kenyon cell using the Mann-Whitney U test but none of the values were found to be significantly different. All source data used in this figure are provided in the Source Data file.

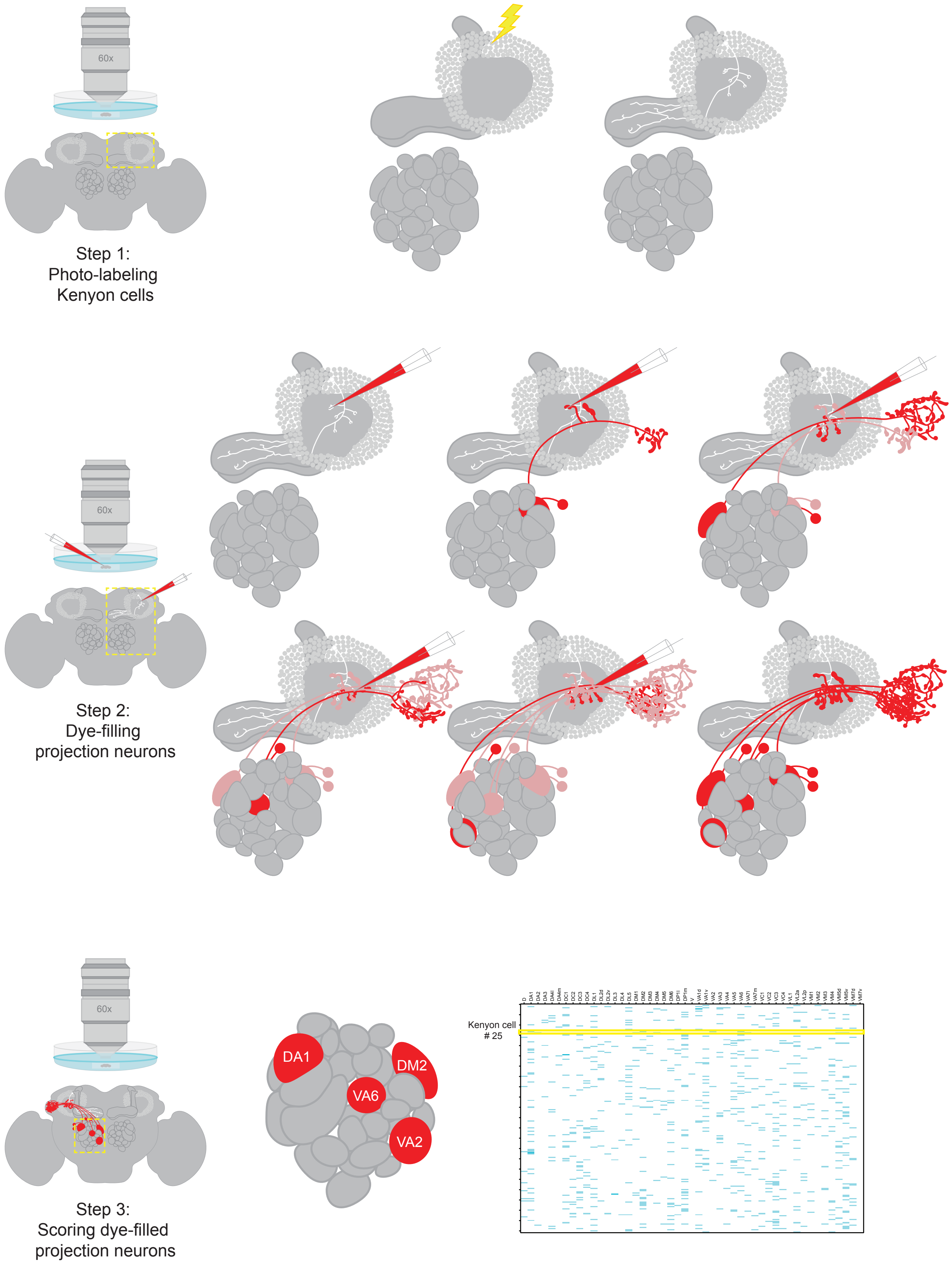

Supplementary Figure 3

Supplementary Figure 3. Mapping technique. Schematic depicting the three-step technique used to map connections between projection neurons and Kenyon cells. Step 1 — Photo-labeling Kenyon cells: The brain of a fly carrying the *nSynaptobrevin-GAL4* and *UAS-photoactivatable-GFP* transgenes is dissected and imaged using 2-photon microscopy (yellow dashed box); a randomly selected Kenyon cell is targeted with high energy light (yellow lightning bolt) such that the photoactivatable-GFP molecules is converted only in that cell; the converted photoactivatable-GFP molecules rapidly diffuse in the Kenyon cell revealing its entire morphology (white). Step 2 — Dye-filling projection neurons: A post-synaptic terminal — or claw — is targeted with an electrode filled with Texas Red dextran dye (red), and, following a short current pulse, the dye is electroporated into the projection neuron connected to that claw; this procedure can be repeated with other claws. Step 3 — Scoring dye-filled projection neurons: The identity of the dye-labeled projection neurons connected to the photo-labeled Kenyon cell can be revealed by visualizing the antennal lobe (yellow dashed box); the glomerular inputs of a given Kenyon cell — in this example the DA1, DM2, VA2 and VA6 glomeruli connecting to Kenyon cell #25 — is reported as a line in a connectivity matrix; each matrix reports the inputs identified for 200 Kenyon cells.

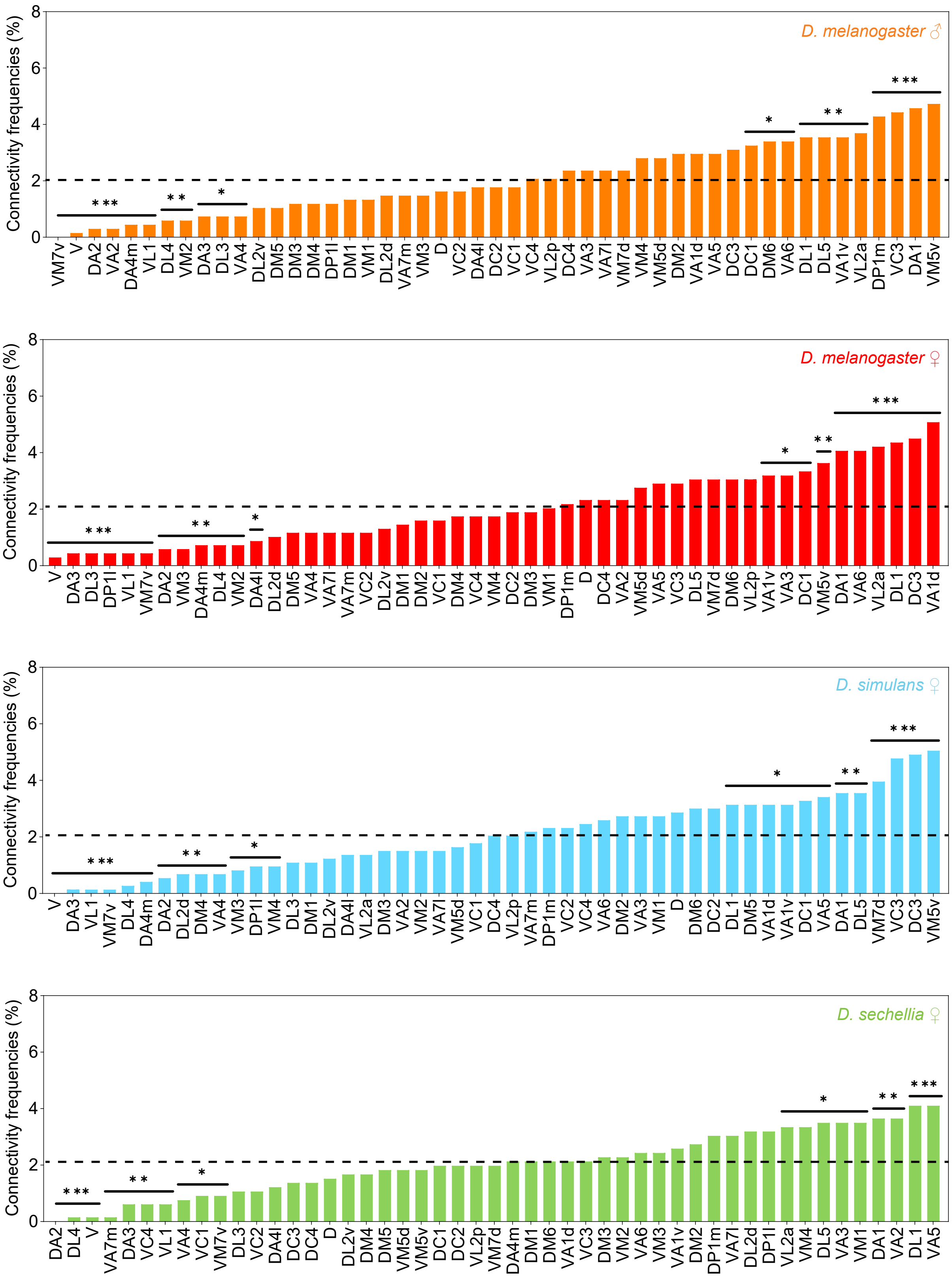

Supplementary Figure 4

Supplementary Figure 4. Non-uniform distribution of connectivity frequencies. The frequencies at which individual glomeruli are connected to Kenyon cells was determined based on the number of connections detected between projection neurons and Kenyon cells in each connectivity matrix reported in Figure 1d (orange: *D. melanogaster* males; red: *D. melanogaster* females; blue: *D. simulans*; green: *D. sechellia*). Glomeruli that are significantly underrepresented or overrepresented are labeled with asterisks (*: p-value < 0.5, **: p-value < 0.01, ***: p-value < 0.001). All source data used in this figure are provided in the Source Data file.

	(Hayashi et al.) *D.mel* female	(Hayashi et al.) *D.mel* female shuffle	(Hayashi et al.) *D.mel* female fixed shuffle	*D.mel* female	*D. mel* female shuffle	*D.mel* female fixed shuffle	*D. mel* male	*D. mel* male shuffle	*D. mel* male fixed shuffle	*D. sec* female	*D. sec* female shuffle	*D. sec* female fixed shuffle	*D. sim* female	*D. sim* female shuffle	*D. sim* female fixed shuffle
(Hayashi et al.) *D.mel* female	0	0.230064	0.084582	0.165927	0.243169	0.170876	0.174747	0.222688	0.191695	0.201030	0.227299	0.211043	0.167408	0.248283	0.176660
(Hayashi et al.) *D.mel* female shuffle	0.230064	0	0.251818	0.241114	0.126355	0.240045	0.252060	0.153742	0.283257	0.239542	0.143807	0.249637	0.262422	0.143807	0.271920
(Hayashi et al.) *D.mel* female fixed shuffle	0.084582	0.251818	0	0.172936	0.262095	0.172478	0.182729	0.242097	0.199343	0.206932	0.249706	0.212911	0.171120	0.265223	0.173369
D.mel female	0.165927	0.241114	0.172936	0	0.255198	0.076578	0.147126	0.233083	0.168658	0.214363	0.219032	0.222641	0.156867	0.251051	0.155649
D. mel female shuffle	0.243169	0.126355	0.262095	0.255198	0	0.250605	0.260611	0.115316	0.285974	0.226794	0.124677	0.241662	0.264687	0.135914	0.274366
D.mel female fixed shuffle	0.170876	0.240045	0.172478	0.076578	0.250605	0	0.160860	0.223744	0.186640	0.202888	0.203326	0.212934	0.173819	0.244101	0.173496
D. mel male	0.174747	0.252060	0.182729	0.147126	0.260611	0.160860	0	0.237657	0.091643	0.216335	0.233334	0.243676	0.160737	0.256017	0.164947
D. mel male shuffle	0.222688	0.153742	0.242097	0.233083	0.115316	0.223744	0.237657	0	0.266267	0.227774	0.111010	0.243587	0.256038	0.126060	0.267556
D. mel male fixed shuffle	0.191695	0.283257	0.199343	0.168658	0.285974	0.186640	0.091643	0.266267	0	0.231651	0.264617	0.258398	0.167737	0.295319	0.171015
D. sec female	0.201030	0.239542	0.206932	0.214363	0.226794	0.202888	0.216335	0.227774	0.231651	0	0.225762	0.092332	0.241181	0.238809	0.253124
D. sec female shuffle	0.227299	0.143807	0.249706	0.219032	0.124677	0.203326	0.233334	0.111010	0.264617	0.225762	0	0.234546	0.253794	0.121173	0.260016
D. sec female fixed shuffle	0.211043	0.249637	0.212911	0.222641	0.241662	0.212934	0.243676	0.243587	0.258398	0.092332	0.234546	0	0.255840	0.247959	0.259560
D. sim female	0.167408	0.262422	0.171120	0.156867	0.264687	0.173819	0.160737	0.256038	0.167737	0.241181	0.253794	0.255840	0	0.265063	0.079589
D. sim female shuffle	0.248283	0.143807	0.265223	0.251051	0.135914	0.244101	0.256017	0.126060	0.295319	0.238809	0.121173	0.247959	0.265063	0	0.280635
D. sim female fixed shuffle	0.176660	0.271920	0.173369	0.155649	0.274366	0.173496	0.164947	0.267556	0.171015	0.253124	0.260016	0.259560	0.079589	0.280635	0

Scale: 0.2, 0.1, 0

Supplementary Figure 5

Supplementary Figure 5. Jensen-Shannon distances. The Jensen-Shannon distances were measured by comparing the distributions in connectivity frequencies observed in the experimental matrices reported in this study (Figure 1d) or in a previous study[29] and the uniform shuffle and biased shuffle matrices; distances are reported as a heat map. The color bar denotes the length of the distances measured. All source data used in this figure are provided in the Source Data file.

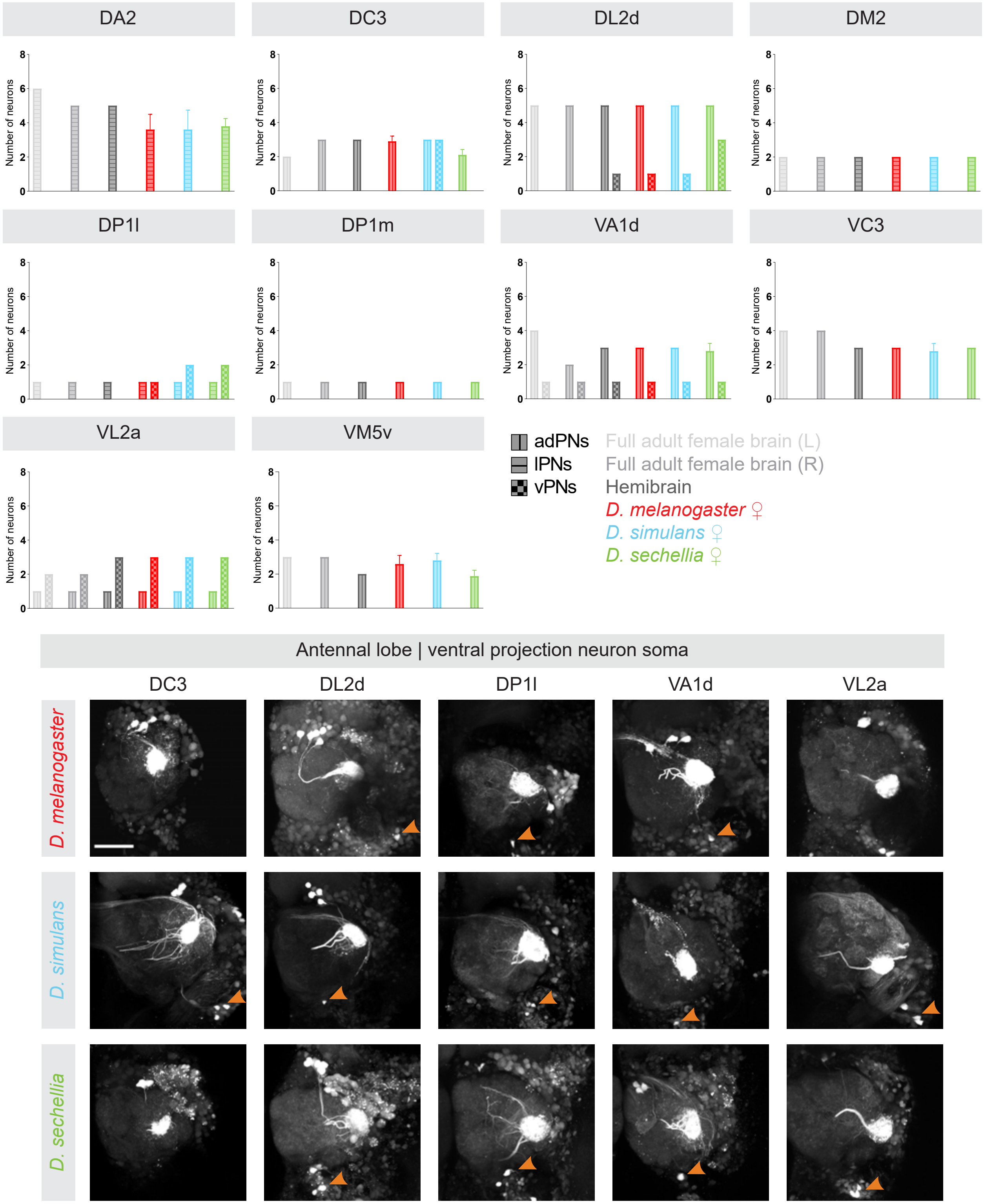

Supplementary Figure 6

Supplementary Figure 6. Number of projection neurons per glomerulus. The number of projection neurons associated with the DA2, DC3, DL2d, DM2, DP1l, DP1m, VA1d, VC3, VL2a, and VM5v glomeruli — divided by types (vertical lines: projection neurons from the anterior-dorsal clusters or adPNs; horizontal lines: projection neurons from the lateral clusters or lPNS; checkers: projection neurons from the ventral clusters or vPNs) — were compared across species based on the data sets collected in this study and the available *D. melanogaster* connectomes[23] (light grey: FAFB connectome; medium grey: FAFB connectome; dark grey: hemibrain connectome). The cell bodies of the DC3, DL2d, DP1l, VA1d, and VL2a projection neurons that are located in the ventral cluster (orange arrows) that were photo-labeled in *D. melanogaster* (upper panels), *D. simulans* (middle panels) and *D. sechellia* (lower panels) are shown (n = 5; standard deviation from mean is shown). Scale bar is 50 µm. All source data used in this figure are provided in the Source Data file.

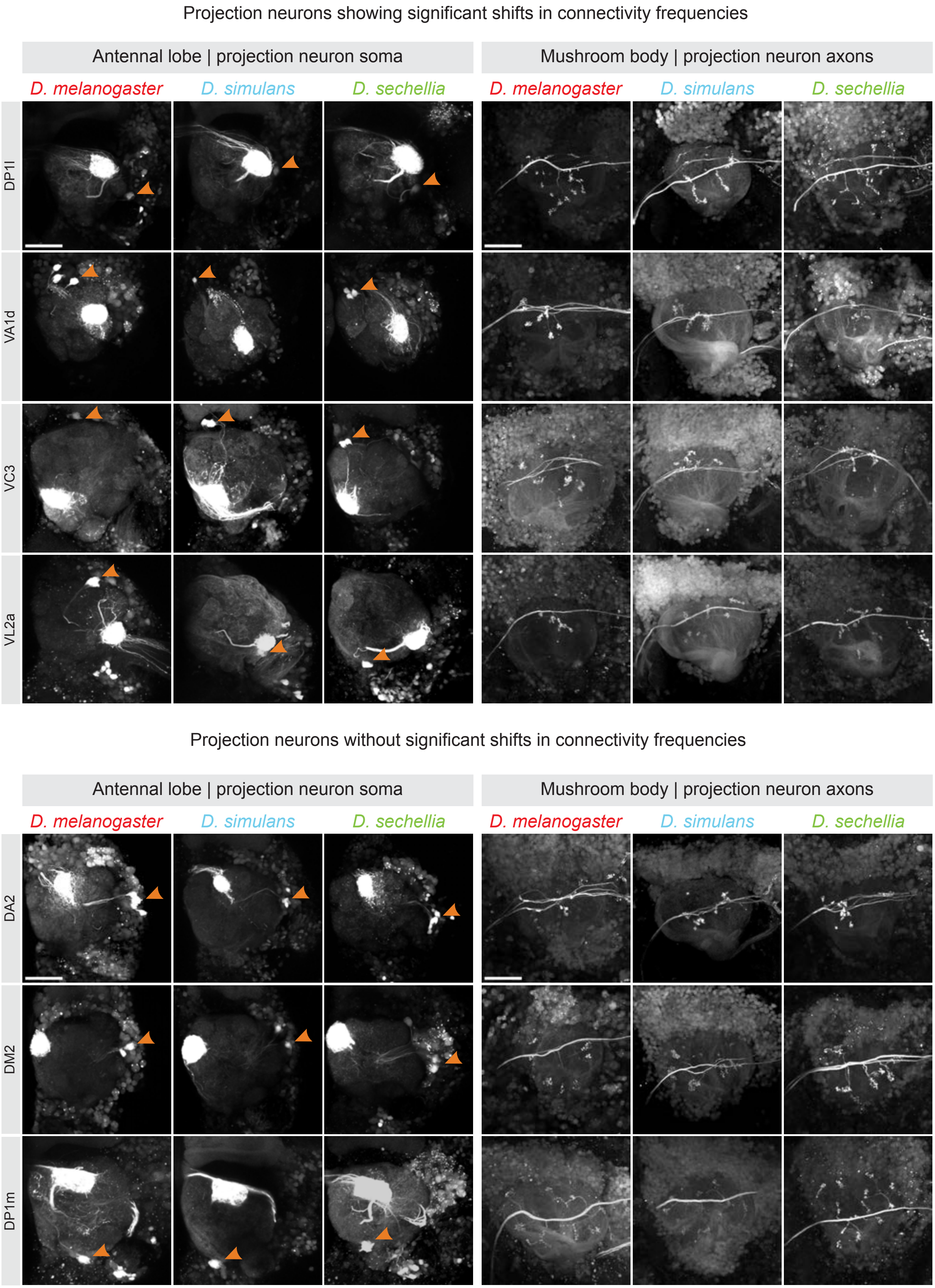

Supplementary Figure 7

Supplementary Figure 7. Morphological features of projection neurons across species. Projection neurons showing significant shifts in connectivity frequencies (DP1l, VA1d, VC3 and VL2a) were analyzed as well as projection neurons showing no such shifts (DA2, DM2 and DP1m). Projection neurons were photo-labeled in *D. melanogaster*, *D. simulans* and *D. sechellia*; the location of the cell bodies of the photo-labeled neurons relative to the antennal lobe (orange arrows) and the morphology of the axonal termini that these neurons extend in the mushroom body were imaged. Scale bar is 50 μm. See Table 1 for quantifications. All source data used in this figure are provided in the Source Data file.

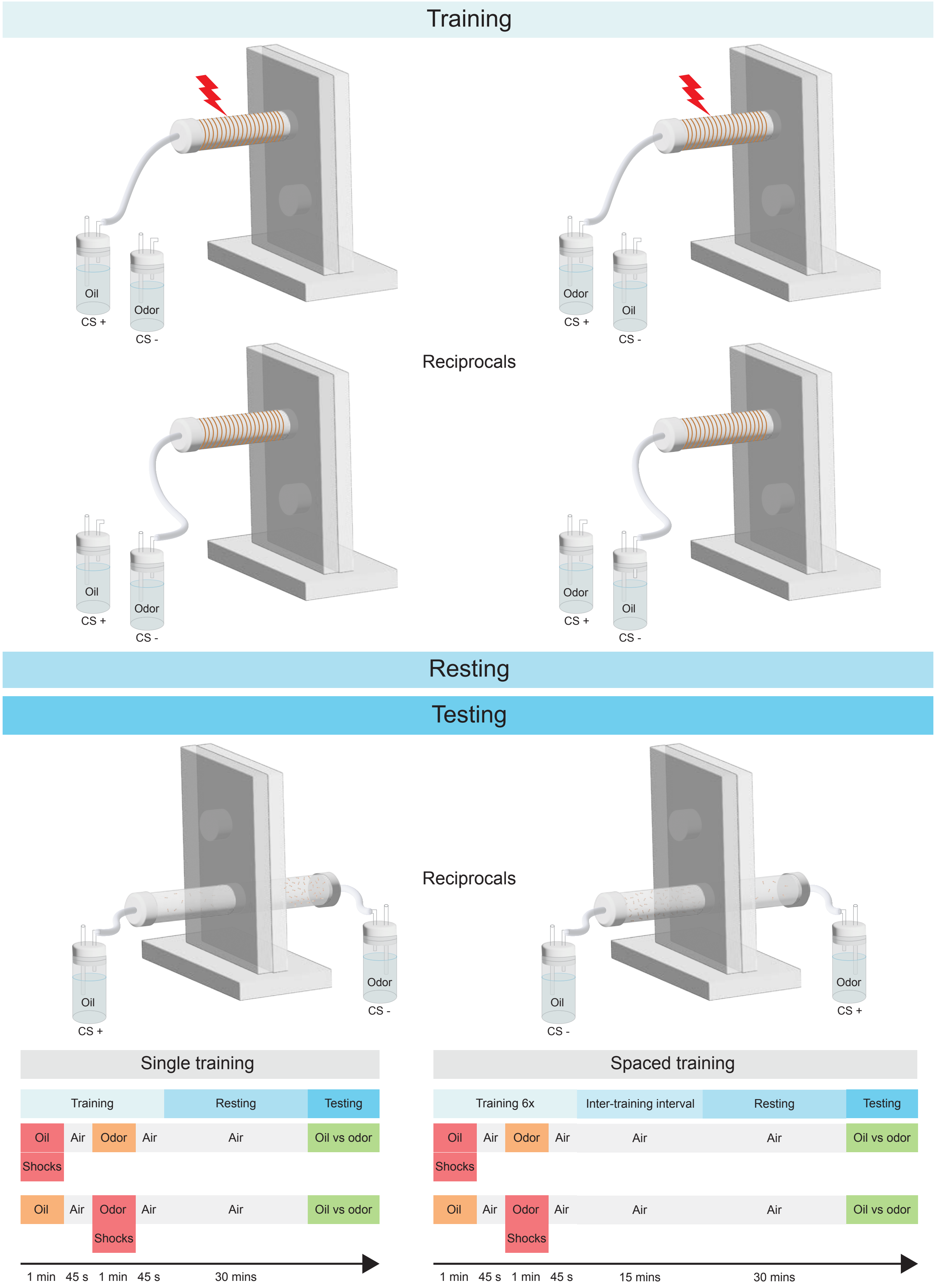

Supplementary Figure 8

Supplementary Figure 8. Aversive learning paradigm. Flies were trained in an aversive learning paradigm using two different protocols. During the training phase, flies were presented with either an odor (farnesol, hexanoic acid, 4-methylcyclohexanol, 3-octanol, 2,3-butanedione or isopentyl acetate) diluted in mineral oil or pure mineral oil while receiving electric shocks (CS+); soon after, flies were exposed to the other stimulus without experiencing electric shocks (CS-); in the reciprocal experiment, the reverse pairing was performed. Flies were allowed a resting phase of 30 minutes. During the testing phase, the preference of flies to seek out the CS+ over the CS- was measured and reported as a Performance Index. Flies were trained using a protocol that included a single regimen of twelve electric shocks or a protocol that included six spaced regimens of electric shocks.

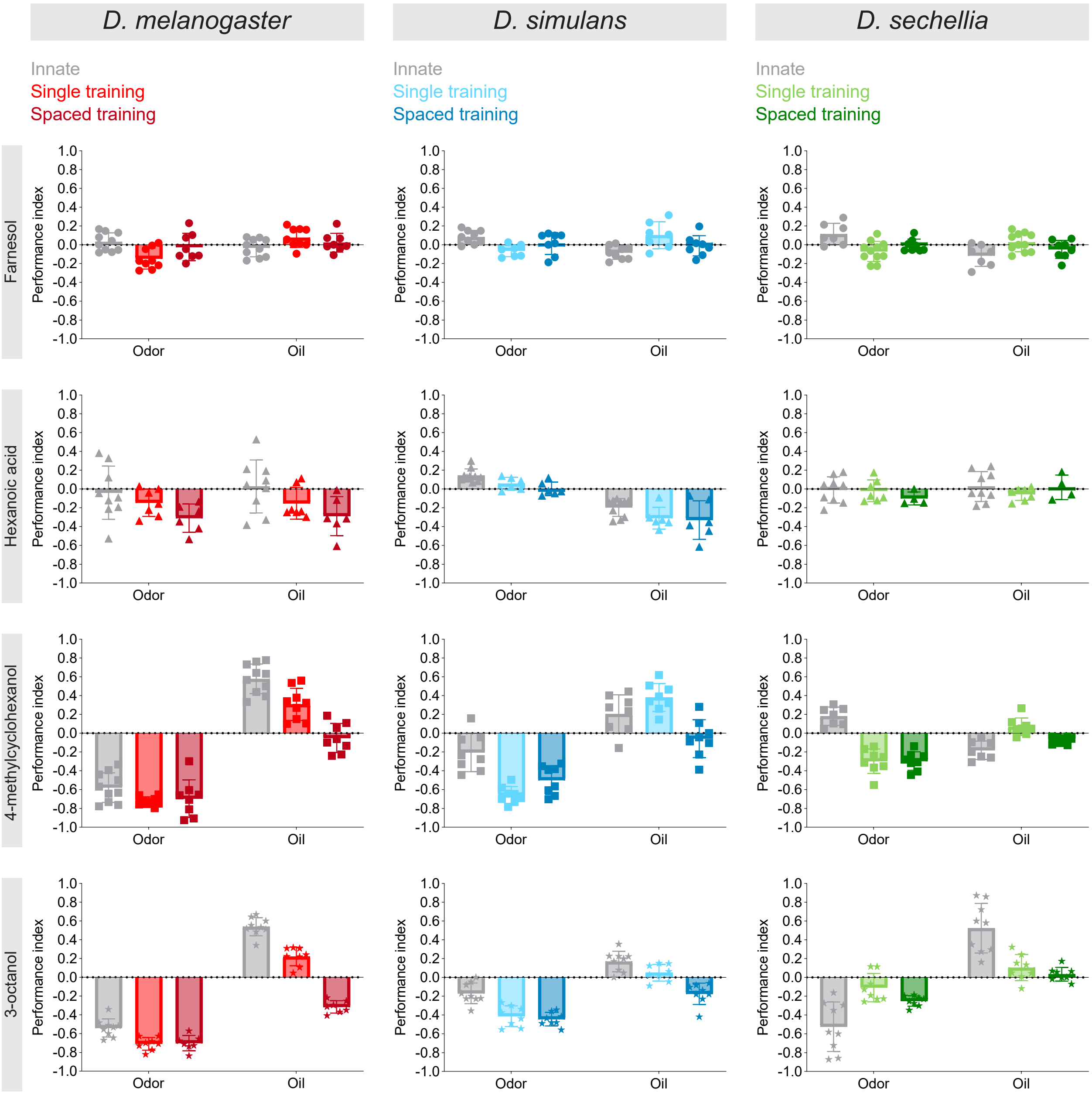

Supplementary Figure 9

Supplementary Figure 9. Single and spaced training. Flies (*D. melanogaster*: red and left column; *D. simulans*: blue and middle column; *D. sechellia*: green and right column) were trained to associate an odor (hexanoic acid: triangles, farnesol: circles, 4-methylcyclohexanol: squares or 3-octanol: stars) with punitive electric shocks using a single regimen of shocks (bright red, blue and green) or six regimens of shocks (dark red, blue and green) and learning was measured as a Performance Index; the response of flies to the stimulus before training is shown (gray). Plots showing the Performance Indices obtained for the odor-pairing (odor) and the reciprocal training (mineral oil) are shown (*n* ≥ 7; standard deviation from mean is shown). All source data used in this figure are provided in the Source Data file.

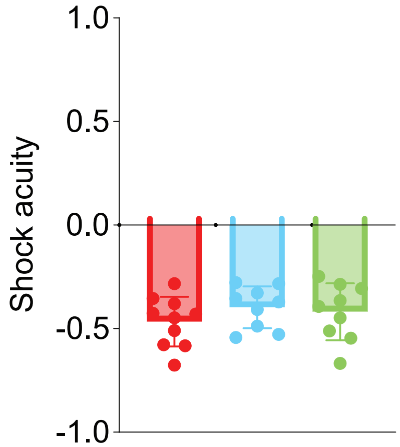

D. melanogaster ♀

D. simulans ♀

D. sechellia ♀

Supplementary Figure 10

Supplementary Figure 10. Shock acuity. Shock acuity was measured by allowing flies (*D. melanogaster*: red; *D. simulans*: blue; *D. sechellia*: green) to choose between a chamber lined with a copper grid onto which 90V electric shocks were delivered every five seconds over the course of one minute. The statistical significance, or *p*-value, was measured using the Anova test but none of the values were found to be significantly different ($n \geq 9$; standard deviation from mean is shown). All source data used in this figure are provided in the Source Data file.

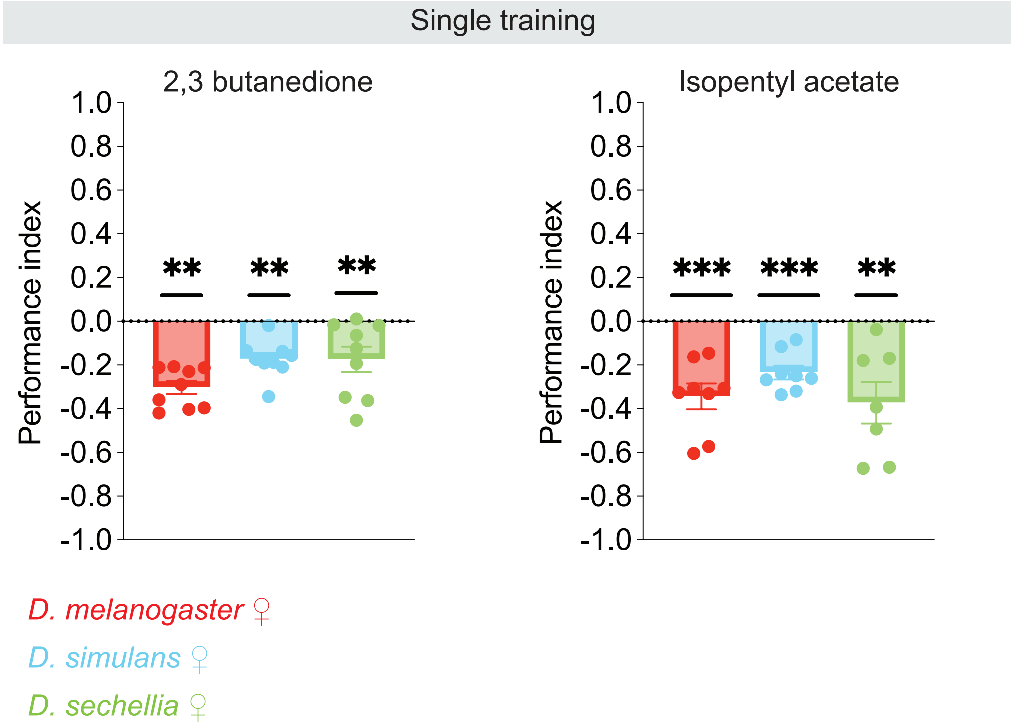

Supplementary Figure 11

Supplementary Figure 11. ***D. sechellia*** **can learn odors that activate multiple receptors.** Flies (*D. melanogaster*: red; *D. simulans*: blue; *D. sechellia*: green) were trained to associate 2,3 butanedione or isopentyl acetate with punitive electric shocks using a single regimen of shocks and learning was measured as a Performance Index ($n \geq 8$); the Performance Indices obtained for the odor-pairing and the reciprocal pairing was averaged. The statistical significance, or *p*-value, was measured using the sample t test using 0 as the hypothetical mean (**: p-value < 0.01, ***: p-value < 0.001; $n \geq 7$; standard deviation from mean is shown). All source data used in this figure are provided in the Source Data file.
