# Supplementary material for: Evolution of connectivity architecture in the Drosophila mushroom body
Source: Nat Commun. 2024 Jun 7;15:4872. doi: 10.1038/s41467-024-48839-4 (PMC11161526; doi:10.1038/s41467-024-48839-4)
Supplement: Supplementary file 3 — Description of Additional Supplementary Files [file 41467_2024_48839_MOESM3_ESM.pdf]

Supplementary Data 1. Comparison of glomerular volumes across species. Individual glomeruli were reconstructed and their volumes were measured in *D. melanogaster*, *D. simulans* and *D. sechellia* (*n* = 3 for each species, standard deviation from mean is shown). See Supplementary Figure 1 for antennal lobe reconstructions. All source data used in this table are provided in the Source Data file.

Supplementary Data 2. Comparison of connectivity frequencies across species. The frequencies at which projection neurons innervating a given glomerulus are connected to Kenyon cells were measured based on the number of connections detected in the connectivity matrices shown in Figure 1d. The statistical significance, or *p*-value, was measured when comparing connection frequencies across species using a binomial test; red text indicates *p*-value < 0.01. All source data used in this table are provided in the Source Data file.
